# A Self-Managed System for Automated Assessment of UPDRS Upper Limb Tasks in Parkinson’s Disease

**DOI:** 10.3390/s18103523

**Published:** 2018-10-18

**Authors:** Claudia Ferraris, Roberto Nerino, Antonio Chimienti, Giuseppe Pettiti, Nicola Cau, Veronica Cimolin, Corrado Azzaro, Giovanni Albani, Lorenzo Priano, Alessandro Mauro

**Affiliations:** 1 Institute of Electronics, Computer and Telecommunication Engineering, National Research Council, 10129 Torino, Italy; roberto.nerino@ieiit.cnr.it (R.N.); antonio.chimienti@ieiit.cnr.it (A.C.); giuseppe.pettiti@ieiit.cnr.it (G.P.); 2 Department of Electronics, Information and Bioengineering, Politecnico di Milano, 20133 Milano, Italy; nicola.cau@polimi.it (N.C.); veronica.cimolin@polimi.it (V.C.); 3 Department of Neurology and NeuroRehabilitation, Istituto Auxologico Italiano, IRCCS, S. Giuseppe Hospital, 28824 Piancavallo, Italy; c.azzaro@auxologico.it (C.A.); g.albani@auxologico.it (G.A.); lorenzo.priano@unito.it (L.P.); alessandro.mauro@unito.it (A.M.); 4 Department of Neurosciences, University of Turin, 10124 Torino, Italy

**Keywords:** Parkinson’s disease, UPDRS, movement disorders, human computer interface, RGB-Depth, hand tracking, automated assessment, machine learning, at-home monitoring

## Abstract

A home-based, reliable, objective and automated assessment of motor performance of patients affected by Parkinson’s Disease (PD) is important in disease management, both to monitor therapy efficacy and to reduce costs and discomforts. In this context, we have developed a self-managed system for the automated assessment of the PD upper limb motor tasks as specified by the Unified Parkinson’s Disease Rating Scale (UPDRS). The system is built around a Human Computer Interface (HCI) based on an optical RGB-Depth device and a replicable software. The HCI accuracy and reliability of the hand tracking compares favorably against consumer hand tracking devices as verified by an optoelectronic system as reference. The interface allows gestural interactions with visual feedback, providing a system management suitable for motor impaired users. The system software characterizes hand movements by kinematic parameters of their trajectories. The correlation between selected parameters and clinical UPDRS scores of patient performance is used to assess new task instances by a machine learning approach based on supervised classifiers. The classifiers have been trained by an experimental campaign on cohorts of PD patients. Experimental results show that automated assessments of the system replicate clinical ones, demonstrating its effectiveness in home monitoring of PD.

## 1. Introduction

Parkinson’s Disease (PD) is a chronic neurodegenerative disease characterized by a progressive impairment in motor functions with important impacts on quality of life [1]. Clinical assessment scales, such as Part III of the Unified Parkinson’s Disease Rating Scale (UPDRS) [2], are employed by neurologists as a common basis to assess the motor impairment severity and its progression. In ambulatory assessments, the patient performs specifically defined UPDRS motor tasks that are subjectively scored by neurologists on a discrete scale of five classes of increasing severity. For upper limb motor function, the specific UPDRS tasks are Finger Tapping (FT), Opening-Closing (OC) and Pronation-Supination (PS) of the hand. During the assessment process, specific aspects of the movements (i.e., amplitude, speed, rhythm, and typical anomalies) are qualitatively and subjectively evaluated by neurologists to produce discrete assessment scores [3].

On the other hand, a quantitative, continuous and objective scoring of these tasks is desirable because the reliable detection of minimal longitudinal changes in motor performance allows for a better adjustment of the therapy, reducing the effects of motor fluctuations on daily activities and avoiding long term complications [4,5]. Currently, these goals are limited by costs, granularity of the UPDRS scale, intra and inter-rater variability of clinical scores [6].

Another desirable feature is the automation of the assessment, because it opens the possibility to monitor the motor status changes of PD patients more frequently and at home, reducing both the patient discomfort and the costs, so improving the quality of life and the disease management. Several proposed solutions, toward a more objective and automated PD assessment at home, mainly employ wearable and optical approaches [5,7], and make use of the correlation existing between the kinematic parameters of the movements and the severity of the impairment, as assessed by UPDRS [3,8,9,10].

Solutions for upper limb task assessment based on hand-worn wireless sensors (i.e., accelerometers, gyroscopes, resistive bands) [8,9,10,11,12] do not suffer from occlusion problems, but are more invasive for motor-impaired people with respect to optical approaches and, more importantly, with an invasiveness that can affect motor performance. Some optical-based approaches for hand tracking in the automated assessment of upper limb tasks of UPDRS have been recently proposed based on video processing [13], RGB-Depth cameras combined with colored markers [14], video with the aid of reflective markers [15], and bare hand tracking by consumer-grade depth-sensing devices [16,17,18].

In this context, tracking accuracy is an important requirement for a reliable characterization of motor performance based on kinematic features. The Microsoft Kinect^®^ device [19,20] accuracy has been assessed in the PD context, resulting restricted to timing characterization of hand movement, due to the limitations of its hand model [21]. The Leap Motion Controller^®^ (LMC) [22] and the Intel RealSense^®^ device family [23] offer more complex hand models and better tracking accuracy. In particular, the LMC is imposing itself as a major technological leap and it is widely used in human computer interaction [24,25] and rehabilitation applications [26,27]. LMC tracking accuracy has been evaluated by a metrological approach mainly in quasi-static conditions [28,29], showing submillimeter accuracy, even if limited to a working volume. In healthcare applications, such as visually guided movements and pointing tasks, the accuracy in moderate dynamic condition is significantly lower, in comparison with the gold-standard motion capture systems [30,31,32]. The Intel RealSense^®^ device family has been characterized for close-range applications [33], even if its hand tracking accuracy has been evaluated only for specific applications [19,34,35]. Its tracking firmware shows similar limitations in tracking fast hand movements, as discussed in [19,31] and in this paper. Furthermore, the typical short product life span of these devices and of the related software support [36] warns against solutions too dependent on closed and proprietary hardware and software.

Along this line of research, we propose as an alternative a low-cost system for the automated assessment of the upper limb UPDRS tasks (FT, OC, PS) at home. The system hardware is based on light fabric gloves with color markers, a RGB-Depth sensor, and a monitor, while the software implements the three-dimensional (3D) tracking of the hand trajectories and characterizes them by kinematic features. In particular, the software performs the real-time tracking by the fusion of both color and depth information obtained from the RGB and depth streams of the sensor respectively, which makes hand tracking and assessment more robust and accurate, even for fast hand movements. Moreover, the system acts at the same time as a non-invasive Human Computer Interface (HCI), which allows PD patients the self-management of the test execution. The automated assessment of FT, OC, PS tasks is performed by a machine learning approach. Supervised classifiers have been trained by experiments on cohorts of PD patients assessed, at the same time, by neurologists and by the system, and are then used to assess new PD patient performance. An important feature of our solution is that it does not rely on any particular hardware or firmware; it only assumes the availability of RGB and depth streams at reasonable frame rate. 

The rest of the paper is organized as follows: first, the hardware and software of the automated assessment system are described, along with the details of its HCI. Then, the experimental setup for the comparison of the tracking accuracy [37] of the HCI respect to consumer devices is detailed. The experiments on PD patients, the kinematic feature selection and the supervised classifier training for the automated assessment are described in the following section. Finally, the Results section presents the tracking accuracy of our HCI with respect to consumer hand tracking devices, the discriminatory power of the selected parameters for motor task classification, and the classification accuracy obtained by the trained classifiers for upper limb UPDRS task assessment.

The overall results for the user usability of the HCI and on the accuracies in the automated assessment of upper limb UPDRS tasks demonstrate the feasibility of the system in at-home monitoring of PD.

## 2. Hardware and Software of the PD Assessment System

### 2.1. System Setup

**Figure 1:**
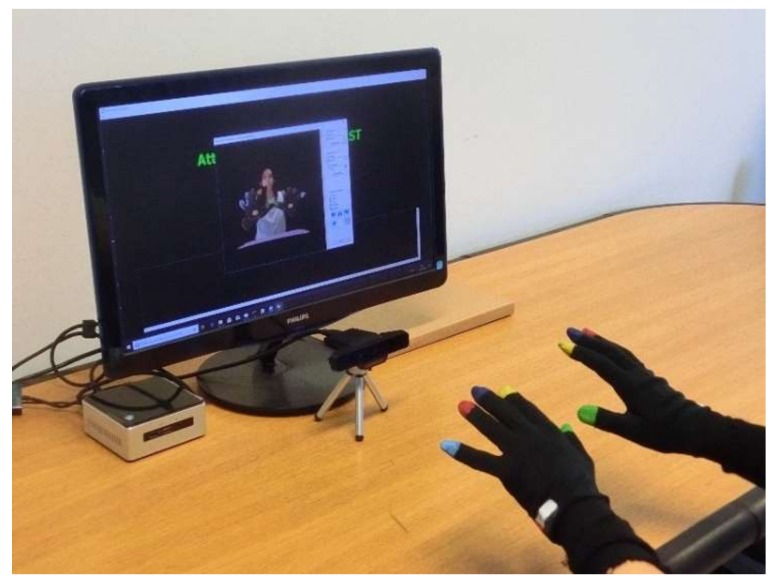
System for the upper limbs analysis: RGB-Depth camera (Intel RealSense^®^ SR300); NUC i7 Intel^®^ mini-PC; lightweight gloves with color markers.

The system hardware is built around a low-cost RGB-Depth camera, the Intel RealSense SR300^®^, which provides, through its SDK [23], synchronized RGB color and DEPTH streams at 60 frame/sec with resolution of 1280 × 720 and 640 × 480, respectively. The range of depth is from 0.2 m to 1.5 m for use indoors. The SR300 is connected via an USB 3 port to a NUC i7 Intel^®^ mini-PC running Windows^®^ 10 (64×) and equipped with a monitor for the visual feedback of the hand movements of the user. The user equipment consists of black silk gloves with imprinted color markers, which are used both for the gestural control of the system and for task assessments (Figure 1).

The system software is made by custom scripts written in C++ which run on the NUC. The software implements different functionalities: a Human Computer Interface (HCI) based on hand tracking, through the SR300 data stream acquisition, real-time processing and visual feedback; a movement analysis and characterization, through the processing of the fingertip trajectories obtained by the HCI; an automated assessment of the hand movements, through the implementation of trained supervised classifiers.

### 2.2. System Software

#### 2.2.1. Initial Setup for Hand Tracking

At startup of the management software, the system automatically performs an initial setup in which the user is prompted to stay with one hand up and open in front of the camera. During this phase, which lasts only a few seconds, global RGB image brightness adjustment, hand segmentation and color calibration are performed, with the SR300 in manual setting mode. The depth stream is acquired through access to the APIs of the SR300 SDK [23], and it is processed in the OpenCV environment to recover the centroid of the depth points closest to the camera, considered approximately coincident with the hand position [38]. The hand centroid is then used to segment the hand from the background and to define 2D and 3D hand image bounding boxes, both for color and depth images.

A brightness adjustment and a color constancy algorithm are performed to compensate for different ambient lighting conditions. First, the segmented hand RGB streams are converted into the HSV color space, which is more robust to brightness variations [38]. Afterwards, a color constancy algorithm is used to compensate for different ambient lighting conditions [39]. For this purpose, the white circular marker on the palm is detected and tracked by the Hough Transform [38] in the HSV stream. The average luminance is evaluated and used to classify the environmental lighting condition as low, normal or high intensity. The average levels of each HSV component are also evaluated to compensate for predominant color components, which can be due to different types of lighting, such as natural, incandescent lamps and fluorescent lamps: their values are used to scale each of the three HSV video sub-streams during the normal operation phase.

Depending on the measured lighting conditions on the glove (low, normal, high intensity), one triplet out of three of HSV threshold values is chosen for the marker segmentation process during the normal operation phase. These three triplets of thresholds were experimentally evaluated for every specific color of the markers in the three glove lighting conditions considered previously.

#### 2.2.2. Continuous Hand and Finger Tracking

**Figure 2:**
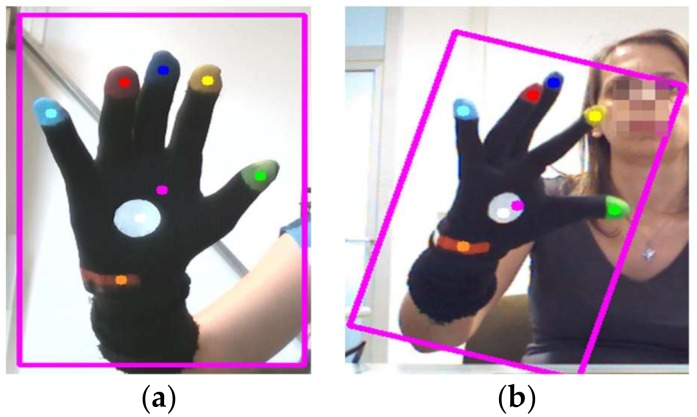
Result of the hand segmentation and the detection of color markers; bounding box of the hand with glove and centroids of the color blobs: (**a**) open hand in static and frontal pose; (**b**) semi-closed hand in dynamic and rotated pose.

During the normal operation phase, the depth stream segmentation described in the setup phase is performed continuously and the 3D position of the hand centroid is used to update the 2D and 3D hand bounding boxes (Figure 2). The color thresholds, selected in the initial setup phase, are used to detect and track the color blobs of all the markers.

To improve performance and robustness, the CamShift algorithm [38] was used for the tracking procedure. Cumulative histograms for glove and color markers are used to define the contours of the hand and each color marker more accurately. The 2D pixels of every color marker area are re-projected to their corresponding 3D points by standard re-projection [38], and their 3D centroids are then evaluated. Each 3D centroid is used as an estimation of the 3D position of the corresponding fingertip. The 3D marker trajectories are then used for movement analysis. The accuracy of the system in the 3D hand tracking of the movements, prescribed for upper limb UPDRS assessment, has been compared to other consumer hand trackers software (LMC and RealSense) and the comparison outcomes are shown in Results section.

#### 2.2.3. Human Computer Interface and System Management

**Figure 3:**
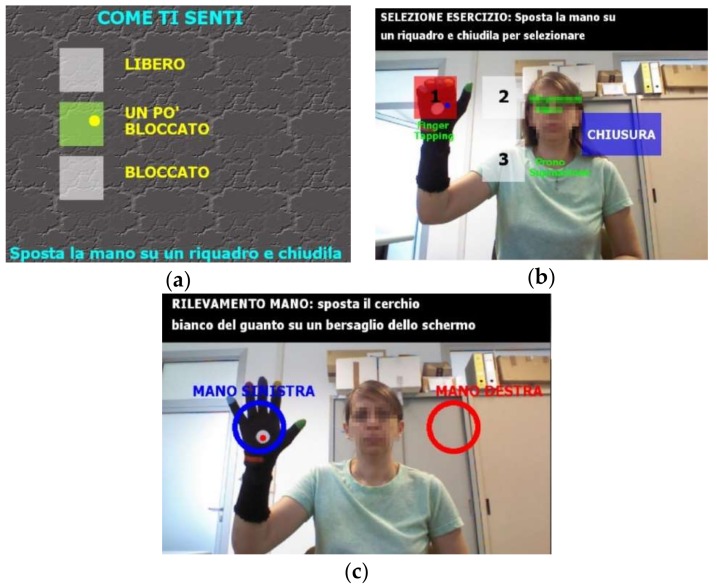
Human computer interface with natural gesture-based interaction: (**a**) patient’s input of the perceived motor impairment condition (low, medium, high) by hand positioning and closing on the menu items; (**b**) selection of the motor test (1, FT; 2, OC; 3, PS) by hand positioning and closing on the menu items (**c**) selection of the hand (SX, DX) involved in the motor test by positioning of the hand inside one of the circular targets.

The hand tracking capability of the system and the graphical user interface (GUI) of the system management software are used to implement a HCI based on gestural interactions and visual feedback, which provides a natural interface suitable for subjects with limited computer skills and with motor impairments. During the assessment tasks session, the user is guided through the GUI menu by video and textual support and can make choices by simple gestures, such as pointing at the menu items to select and closing the hand or fingers to accept (Figure 3).

To remind the user of the possible choices, or when an incorrect sequence of actions takes place, suggestions are displayed as text output on the screen. At every point during the assessment session, the user can stop the session and quit, if tired, to avoid the onset of stress and/or anxiety. The data of the sessions (video of each task execution, user inputs, finger trajectories and assessment scores) are encrypted and recorded on the system hardware to provide remote supervising facilities to authorized clinicians.

## 3. Performance Comparison of the HCI Hand Tracker with Consumer Devices

### 3.1. Experimental Setup

An experimental setup was built to evaluate and compare the accuracy of the HCI, the LMC^®^ and the Intel RealSense^®^ SR300 (Intel Corporation, Santa Clara, CA, USA) [40] in the hand tracking of the FT, OC and PS movements. The comparisons were made by using a DX400 optoelectronic system (BTSBioengineering, Milan, Italy) as gold reference (BTS SMART DX400©, 8 TVC, 100–300 fps) [41].

The LMC hand-tracking device [22] is built around two monochromatic IR cameras and three infrared LEDs, which project patternless IR light in a hemispherical working volume. The IR cameras reliably acquire images of the objects (hands) from 2 cm to 60 cm distance in the working volume, at a frame rate of up to 200 fps. To reduce possible interference among the several Infrared Radiation (IR) light sources of the different devices, the comparison was split into two experiments (Section 3.1.1 and Section 3.1.2): in the first one, the HCI, the LMC and the DX400 were involved; in the second one, the HCI, the RealSense SR300 and the DX400 were involved. In both the experiments, the devices’ accuracies were evaluated by comparison of the movements captured at the same time by the two devices with the DX400 reference system. It should be noted that we compared two SR300 devices; one is a component of the HCI of our system, and the other is an external device whose proprietary hand tracking firmware was to be assessed. In this case, we compared the performance of our tracking software, based on the processing of color and depth map of the SR300 implementing the HCI, with the proprietary one of the external SR300.

The movements were performed in the smallest working volume common to all the devices. Specifically, both the LMC and the SR300 used in the HCI have a working volume delimited by a truncated pyramid boundary, whose apex is centered on the device and whose top and base distances are defined by the reliable operating range. This range is established according to the device specifications [22,40] and the results of other experimental works [29,35], also taking into account a minimum clearance during movements, to avoid collisions. Consequently, we assume a reliable operating range for the LMC controller from 5 to 50 cm, while that of SR300 of the HCI can be safely reduced respect to the device specifications (20 to 120 cm) from 20 to 100 cm, considering the minimum spatial resolution necessary to track the colored marker at the maximum range. Therefore, the reliable working volume for the LMC is about 0.08 m^3^, while that of the HCI is about 0.45 m^3^, which is about six times bigger. Then, the smallest working volume common to all the devices is constrained by the LMC one, and therefore the accuracy evaluations and comparisons are limited to this volume. 

**Figure 4:**
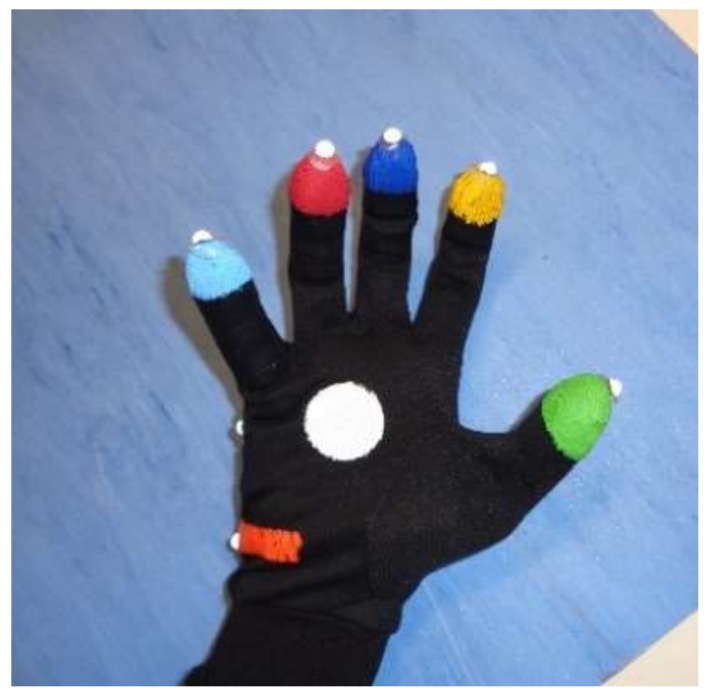
HCI glove with reflective markers on the finger tips.

In the two experiments, five healthy subjects (3 men/2 women) of different heights (from 1.50 to 1.90 m), aged between 25 and 65, were recruited to assess the accuracy of the devices in hand tracking of FT, OC and PS movements. The subjects had no history of neurological, motor and cognitive disorders. The rationale of this choice is to provide a data set of finger trajectories approximately filling the working volume, which are representative both of the specific movements and of the population variability. Moreover, we chose healthy subjects because their movements are faster and of greater amplitude with respect to motor-impaired PD subjects, and therefore they are more challenging for accuracy evaluations. During the experiments, the subjects were seated on a chair facing the HCI and the LMC (or the SR300, in the second experiment), with the chest just beyond the upper range of the working volume. A set of hemispherical retroreflective markers, with diameter of 6 mm, were attached on the fingertips of the subject wearing the HCI glove (Figure 4).

The subjects were told to perform the FT, OC and PS movements as fast and fully as prescribed in UPDRS guidelines [2], with the hand in front of the devices. The movements were performed in different positions, approximately corresponding to the corners and the center of the bounding box of the working volume, with the aid and the supervision of a technician. A total of nine hand positions were sampled in the working volume. The movements were first performed by the right hand in its working volume, then by the left one, after adjusting the chair and subject position to fit its corresponding working volume. The 3D trajectories of the fingers were tracked simultaneously by the HCI and by one of the other two devices, and were then compared with those captured by the DX400 optoelectronic system.

The different 3D positions of the reflective and colored fingertip markers correspond to a 3D displacement vector with constant norm of about 9 mm between their respective 3D centers. This vector was added to the 3D centers of the colored fingertip markers to estimate the “offset free” colored marker trajectory, which was used for the HCI accuracy estimation. To evaluate the influence of the gloves respect to the bare hand on the commercial system accuracy, we performed two preliminary tests. First, we compared the luminance of the IR images of both the bare hand and gloved one, as obtained from the SDKs of the two devices. Please note that, IR images are used as input for the proprietary hand tracking firmware of the LMC. We found no substantial differences between the IR images of the hand in the two cases; neither in the spatial distribution, nor in the intensity of the luminance. Second, as in [29], we compared the fingertip position of a plastic-arm model, fixed on a stand, in different static locations inside the working volume. In every location, we first put on and then removed the glove from the hand, looking at the differences in the 3D fingertip positions for the two conditions. Since we found position differences below 5 mm, we assumed the glove influence to be approximately negligible.

In both the experiments, we checked for possible IR interference among different devices by switching them on and off in all possible combinations, while keeping the hand steady in various positions around the working volume and looking at possible data missing or variations of tracked positions. A safe working zone of approximately 2 × 2 × 2 m in size was found, where the different devices were not influenced by one another. The devices could almost frontally track the hand movement, without line of sight occlusions. In the safe working volume, the claimed accuracy of the DX400 is 0.3 mm, and all markers were seen, at all times, by at least six of the eight cameras placed in a circular layout and few meters around the working zone. Two calibration procedures were used in the two experiments to estimate the coordinate transformation matrices for the alignment of the local coordinate systems of the different devices to the reference coordinate system of the DX400 (Section 3.1.1 and Section 3.1.2).

The devices have different sampling frequencies: a fixed sampling rate of 100 sample per second for the DX400, an almost stable sampling rate of 60 sample per second for the SR300, and a variable sampling rate, which cannot be set by the user, for the LMC, ranging from 50 to 115 samples per second in our experiments. Consequently, the 3D trajectory data were recorded and resampled by cubic spline interpolation at 100 samples per second to compare the different 3D measures at the same time. To compare the accuracy of the different tracking devices, we used the simple metrics developed in [37], which provides a framework for the comparison of different computer vision tracking systems (such as the devices under assessment) on benchmark data sets. With respect to [31], where the standard Bland-Altman analysis was conducted to assess the validity and limits of agreement for measures of specific kinematic parameters, we prefer to adopt the following more general approach and not to define, at this point, which kinematic parameters will be used to characterize the movements.

Consider two trajectories *X* and *Y* composed of 3D positions at a sequence of time steps *i*. According to [37], we use the Euclidean distance *d_i_* between two samples positions *x_i_* and *y_i_* at time step *i* as a measure of the agreement between the two trajectories at time *i*. The mean D_mean_ of these distances *d_i_* provides quantitative information about the overall difference between *X* and *Y*. Here we identify *X* trajectory as measured by the DX400 reference system, and the distances *d_i_* can be interpreted as positional errors. Then, as in [37], we adopt the mean D_MEAN_, the standard deviation SD, and the maximum absolute difference MAD = |*d_i_*|_max_ of the *d_i_* sequence as useful statistics for describing the tracking accuracy. Furthermore, we note that, for the tracking accuracy evaluation of the FT, OC and PS movements, the absolute positional error in the working volume is not important; the correctly performed hand movements are necessarily circumscribed to a small bounding box positioned at the discretion of the subject in the working space.

On the other hand, we know the device measurements are subject to depth offsets increasing with the distance from the device [33]. For this reason, some pairs of trajectories may be very similar, except for a constant difference in some spatial direction; that is, an average offset vector $\bar{d}$ (translation) could be present between the trajectories. Since this offset vector is not relevant for characterizing the movements, we subtract it from the *d_i_* sequence before evaluating the accuracy measures [37], (p. 4). The accuracies were evaluated comparing the finger trajectories measured at the same time by one device and the corresponding one measured by the DX400. Only the trajectory parts falling in the working volume were considered in the comparison. The final measure of the device accuracy is obtained by the average values of the D_MEAN_ and the SD evaluated for all the trajectories captured by the device in the working volume, while for the MAD value the maximum over all the trajectories in working volume is considered.

Custom C++ scripts were developed for both the experiments to collect the data through the SDK APIs of the devices, and custom Matlab^®^ scripts (Mathworks Inc, Natick, MA, USA) were developed to perform the alignment of the finger trajectory data from different devices into the common reference frame of the DX400, and to evaluate accuracy measures (see Table 1 and Table 2).

#### 3.1.1. Leap Motion and HCI Setup

The LMC was positioned facing the subject (the *Y* axis of the LMC reference system was pointing to the subject’s hand) at about 10 cm away from the closest distance of the hand in the working volume, and it was firmly attached on a support to avoid undesired movements of the device. The RGB-Depth sensor of the HCI was placed 10 cm beyond and above the LMC, to avoid direct interferences with the LMC and to allow the maximum overlapping of the working volumes of the two sensors.

An external processing unit (Asus laptop Intel Core i7-8550U, 8 MB Cache) was used to run the scripts accessing the LMC proprietary software (LMC Motion SDK, Core Asset 4.1.1) for real-time data acquisition and logging. The final information provided by the scripts was the positions over time of 22 three-dimensional joints of a complex hand model, which includes fingertips. The LMC Visualizer software was used to monitor, in real time, the reliability of the acquisitions.

**Figure 5:**
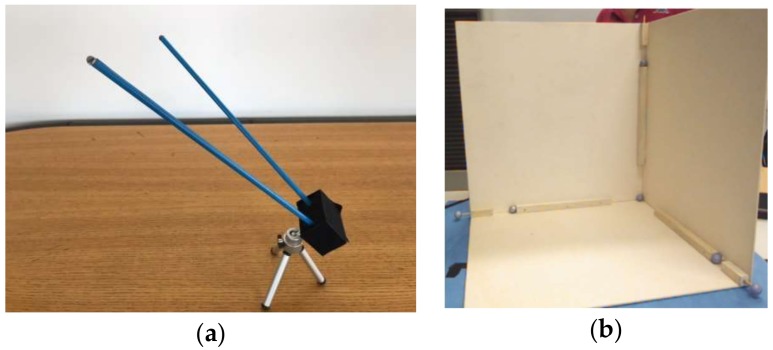
Calibration tools: (**a**) V-shaped tool; (**b**) Dihedral tool.

The calibration procedure, used to estimate the alignment transformation between the LMC controller and the DX400 coordinate frames, makes use of the same special V-shaped tool (Figure 5a) as in [29], which consists of two wooden sticks fixed on a support and two reflective markers fixed on the stick tips. The tool was moved around the working space and tracked both by the LMC and the DX400. The alignment transformation was estimated as the transformation (roto-translation) which best aligns the two set of tracking data by the two devices.

A different approach was used to estimate the alignment transformation between the coordinate frames of the SR300 device of the HCI and the DX400. A dihedral target, made of three planes orthogonal to each other, was built (Figure 5b). Seven reflective markers were attached at its center and along the axis at a fixed distance from the origin. The depth maps of the SR300 device of the HCI were processed to extract the planes, and to estimate the dihedral plane intersections and their origin in the local coordinate system [42]. The positions of the reflective marker on the plane intersections were measured by the DX400, in the global coordinate system. The alignment transformation was then estimated as the transformation which best aligns the two sets of data tracked by the two devices.

#### 3.1.2. Intel SR300 and HCI Setup

We refer to Section 2.1 for a brief description of the RealSense SR300 features. In this experiment, the Intel SR300 was positioned facing the subject at about 20 cm away from the closest distance of the hand in the working volume, and it was firmly attached on a support to avoid undesired movements. The device transmitted 3D position data of hand and fingertips to the Asus laptop PC (Section 3.1.1) running specifically developed C++ scripts interfacing the SR300 SDK APIs [23] for real-time data acquisition and logging. The same approach described in Section 3.1.1 was used to estimate the alignment transformation between the coordinate frames of the SR300 device respect to the DX400. We note, as in Section 3.1, that we compared two SR300 devices; one is a component of the HCI of our system, and the other is an external device whose proprietary hand tracking firmware was to be assessed.

## 4. Automated Assessment of Upper Limb UPDRS Tasks

### 4.1. Clinical Data Acquisition

A cohort of 57 PD patients (37 men/20 women) was recruited to perform the FT, OC and PS tasks (UPDRS Part III, items 3.4, 3.5, 3.6) under the supervision of a neurologist expert in movement disorders and PD. The patients were chosen according the UK Parkinson’s Disease Society Brain Bank Clinical Diagnostic standards and met the following criteria: Hoehn and Yahr score (average 2.1, min 1, max 4); age 45–80 years; disease duration 2–29 years. Patients were excluded if they had had previous neurosurgical procedures, tremor severity >1 (UPDRS-III severity score), or cognitive impairment (Mini–Mental State Examination score <27/30). All the patients were allowed to take their routine PD medications. Another cohort of 25 healthy controls (10 men/15 women), aged between 55 and 75, was recruited. Healthy Controls (HC) had no history of neurological, motor and cognitive disorders. Informed consent was obtained in accordance with the Declaration of Helsinki (2008). The study’s protocol was approved by the Ethics Committee of the Istituto Auxologico Italiano (Protocol n. 2011_09_27_05).

The PD subjects were seated in front of the system, wearing the gloves of the HCI: their hand movements were acquired during UPDRS tasks execution and then analyzed by the system and scored by the neurologist at the same time. Some of the visual features of interest for the neurologist are rate, rhythm, amplitude, hesitations, halts, decrements in amplitude and speed, as indicated by the UPDRS. The neurologist classified the performances of the PD cohort in four UPDRS levels, i.e., 0 (normal), 1 (slightly impaired), 2 (mildly impaired), 3 (moderately impaired). No patient of the PD cohort was assessed as UPDRS 4 (i.e., severely impaired). The HC subjects performed FT, OC and PS tasks in the same environmental conditions and with the same setup of PD subjects. The system recorded the videos and the 3D trajectories of the PD and HC cohort performances, along with the assigned UPDRS scores of the PD cohort for each single task.

### 4.2. Movement Characterization by Kinematic Features

As mentioned in the Introduction, the automatic assessment of UPDRS tasks makes use of the well-established correlation existing between kinematic features of the movements and clinical UPDRS scores. An initial set of kinematic parameters, estimated from the 3D trajectories, were used to characterize the hand movements; these parameters are closely related to those features of the movement that are implicitly used by neurologists to score the motor performance of the patient.

The initial sets of kinematic parameters, considered to characterize the hand movements during the FT, OC and PS tasks, consisted of about twenty parameters per task. These parameters are closely related to those features of the movement that are implicitly used by neurologists to score the motor performance of the patient, as described in the Introduction. These initial sets could potentially include irrelevant and redundant parameters which can hide the effects of clinically relevant parameters and reduce the predictive power of the classifiers. Among the most used feature selection (FS) algorithms in machine learning, the Elastic Net (EN) [43] was chosen to reduce the initial parameter sets to the most discriminative subsets. EN is a hybrid of Ridge regression and LASSO regularization. EN encourages a grouping effect on correlated parameters which tends to be in or out of the model together. In contrast, the LASSO tends to select only one variable from the group, removing from the model the other ones. This behavior can generate incorrect models with our set of parameters, that address similar kinematic features and tend to be moderately correlated. In fact, we found this by inspection of the cross-correlation matrices of the parameters evaluated from the FT, OC and PS datasets. The EN selection procedure we used is based on the Matlab^®^ implementation (lasso function with α parameter). The parameter α (0 ≤ α ≤ 1) controls the function behavior between a Ridge or a LASSO regression.

A dimensionality reduction of the parameter space was performed on the data sets by Principal Component Analysis (PCA), retaining the 98% of the data information content. The sets of parameters which contribute more to the eigenvectors of the PCA representation are compared with those selected by the EN, to check for possible inconsistencies. Depending on the value of α, the number of elements in the selected sets can change, even if the most important parameters remain the same. To stress the parameter correlation with UPDRS scores, the final set of selected parameters are chosen starting from the best ones among those of the EN sets, and taking those having absolute values of the Spearman’s correlation coefficient ρ greater than 0.3, at a significance level *p* < 0.01. The selected parameters for FT, OC and PS are shown, respectively, in Table 3, Table 4 and Table 5 of the Results section. To avoid biasing the results by the different scaling of the parameters not related to clinical aspects, the PD parameters *p*_i PD_ were normalized by the corresponding average parameter values of the HC subjects *p*_i HC_ (Equation (1)); as expected, the average values for healthy subjects are always better than the *p*_i PD_ ones.

*p*_i PD Norm_ = *p*_i PD_/*p*_i HC_, (1)

The normalized parameters of Table 3, Table 4 and Table 5 are able to discriminate the different UPDRS classes for the FT, OC and PS tasks, highlighting the increasing severity of motor impairment by the corresponding increasing of their values. This is confirmed by the radar graphs of the mean values of the kinematic parameters versus UPDRS severity class, as shown in the Results section.

### 4.3. Automated UPDRS Task Assessment by Supervised Classifiers

Different supervised learning methods were evaluated for the automatic assessment of the FT, OC and PS tasks: Naïve-Bayes (NB) classification, Linear Discriminant Analysis (LDA) [44], Multinomial Logistic Regression (MNR), K-Nearest Neighbors (KNN) [45] and Support Vector Machine (SVM) with polynomial kernel [46]. The NB, LDA, MNR and KNN classifier evaluations were performed in the Matlab^®^ environment using specific toolboxes. The SVM classifiers were implemented with the support of the LIBSVM library package [47], and the SVM kernel parameters optimized using a grid search of possible values. Three specific classifiers, one for each task, were trained for every method using the sets of “selected kinematic parameters vector–neurologist UPDRS score” pairs as input. The leave-one-out and the ten-fold cross validation have been used to evaluate the performance in terms of both accuracy and generalization ability. The accuracies of all the methods were compared both for binary classification (healthy subjects, Parkinsonians) and for multiclass classification (five classes: healthy and four UPDRS classes).

## 5. Results

### 5.1. Hand Tracking Accuracy of the HCI Compared to Consumer Devices

#### 5.1.1. HCI—Leap Motion Tracking Accuracy Comparison

The comparison between the accuracy of the HCI tracker respect to the LMC was made for the FT, OC and PS task movements using the DX400 optoelectronic system as reference. The accuracy of HCI in FT, OC, and PS movement tracking, expressed as average values of the mean D_MEAN_, the standard deviation SD, and the maximum absolute difference MAD (Section 3.1) over the whole set of trajectories are shown in Table 1, while in Table 2, the corresponding values for the LMC are shown.

**Figure 6:**
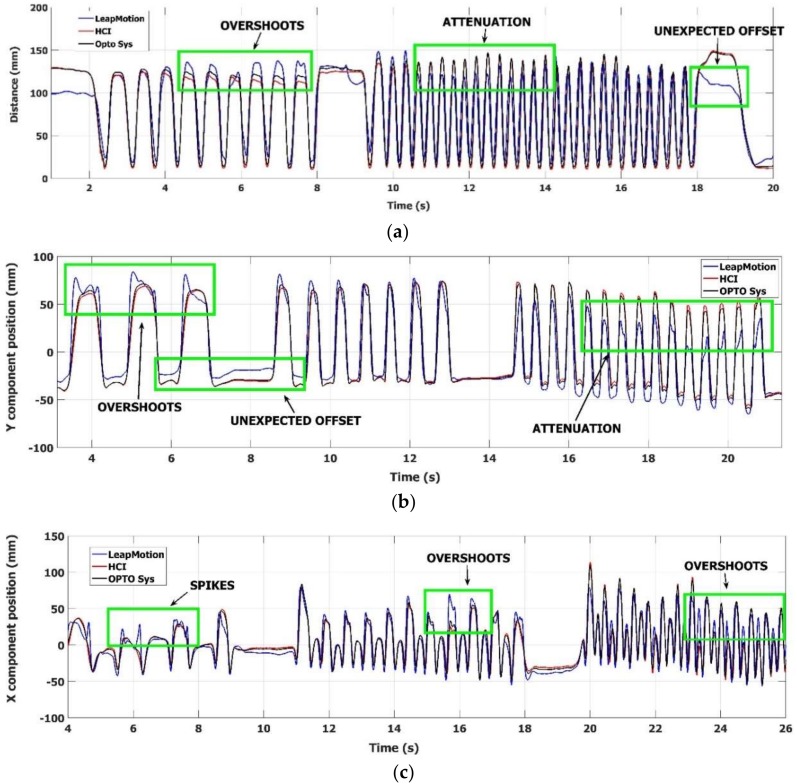
Comparison of the Leap Motion^®^ tracker (blue solid line), the HCI tracker (red solid line) and the optoelectronic system (black solid line) during the execution of the FT, OC and PS motor tasks. The figures show the trajectories simultaneously measured by the three devices: (**a**) distance between thumb and index trajectories during the FT task; (**b**) *Y* component of the index trajectory during the OC task; (**c**) *X* component of the pinky trajectory during the PS task.

**Figure 7:**
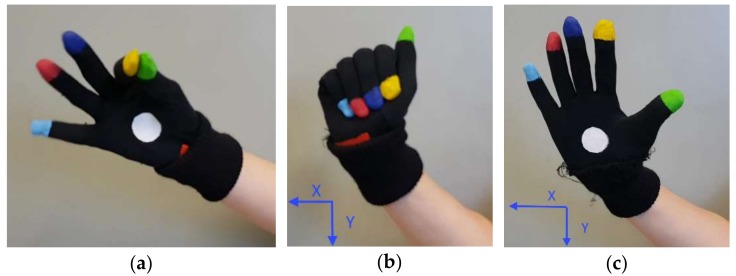
Hand pose without reflective markers during (**a**) FT, (**b**) OC, and (**c**) PS. For OC and PS the reference directions for the components of the movements are shown.

Furthermore, examples of typical trajectories of the FT, OC and PS task movements are shown in Figure 6. Only the trajectories of representative fingers are considered here, to avoid overcrowded graphs. A reference system, whose *X* and *Y* axes are co-planar with the *Z* and *X* axes of the LMC, was chosen as the more convenient to represent at best the differences of the trajectories tracked by the different devices. The reference systems for the *X* and *Y* components of the OC and PS movements are indicated in Figure 7b,c, respectively. In Figure 6a, the distance between the index and thumb fingertips during the FT task movements is plotted, as estimated by the three devices. In Figure 6b, the *Y* component of the index finger movements during the OC task movements is plotted, positive when fingers move down and the hand closes (Figure 7b). In Figure 6c, the *X* component of the pinky finger for the PS task movements is plotted, positive when pinky finger moves left while rotating around the *Y* axis (Figure 7c). As can be seen in Figure 6a, the LMC shows good responsiveness to very quick FT movements (up to 7 FT cycles/sec), but the finger distance shows overshoots for medium-speed FT cycles and attenuations respect to the reference and the HCI values for high-speed FT cycles. Overall, many incomplete finger closures are present in the lower part of Figure 6a, along with unexpected offsets in slow-speed FT cycles. In contrast with LMC, the HCI response follows much better the reference system. Similar problems can be seen in Figure 6b for the OC task, where a good LMC response is obtained for medium-speed OC cycles, while unexpected offsets and attenuations are present for low and high-speed cycles. Again, the HCI response follows much better the reference system measurements.

**Figure 8:**
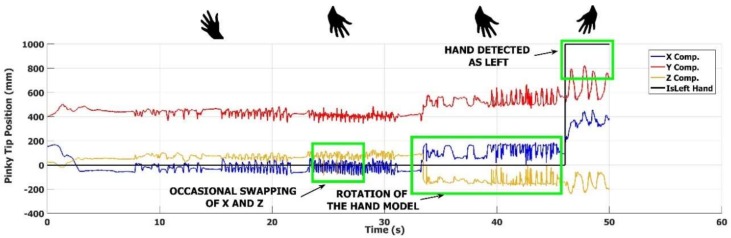
Example of Leap Motion^®^ tracker failures in the pinky tip tracking during a pronation-supination task. The figure shows the three components of the 3D position of the pinky finger during the execution of the movement. After a 20 s period, where the hand pose is correctly estimated, a period of 10 s follows where occasional swapping of the *X* and *Z* components occurs. A persistent incorrect estimation of the hand pose occurs in the following 10 s, ending in the final part of the plot with a misinterpretation of the right-hand movements as performed by the left one.

The PS task is the most challenging one for the hand trackers, because finger velocity could reach more than 2 m/s. In this task the accuracy of HCI tracker is quite satisfactory as compared to the reference. In Figure 6c, the LMC results for the PS task seem better than those for FT and OC tasks, even if offsets and attenuations are still present. However, if we look at Figure 8, in which the three components of the position of the pinky fingertip during PS movements are shown, some instabilities in the LMC tracker become evident.

We remark that, during PS, the hand faces the LMC and performs PS rotating around its main axis *Y* (Figure 7c), while the pinky position moves almost along the *X* axis (Figure 7c). In Figure 8, the first 20 s corresponds to a correct estimation of the hand pose; during rotation, the fingers always point upward along the -*Y* axis (Figure 7c). Some occasional event of swapping of the *X* and *Z* components occurs in the period from 20 to 30 s, as confirmed also from the other finger position values output by the LMC tracker. These events correspond to an inversion of the rotation axis of the PS, from upward to downward, followed by a quick recovering of the correct orientation. Concerning the period from 30 to 45 s, we see that the inversion of the rotation axis of the hand is persistent and evident in the swap of the *X*, *Y* and *Z* components, causing a wrong estimation of the hand movement. In addition, in the final part of the PS period, this behavior leads to a misinterpretation of the performing hand: the task is executed with the right hand but LMC tracker assumes it is performed by the left hand.

Overall, these problems with the LMC tracker limit the feasibility and the accuracy of the kinematic characterization of the hand movements and, consequently, the motor performance assessment based on it.

#### 5.1.2. HCI—RealSense SR300 Tracking Accuracy Comparison

The comparison between the accuracy of the HCI and the SR300 trackers was limited to FT task movements, both because the results provide significant indication on the accuracy and because of Intel’s intention to discontinue the development of the hand tracking part of the camera firmware [40]. The accuracy of HCI in FT movement tracking, expressed as the average values of the mean D_MEAN_, the standard deviation SD, and the maximum absolute difference MAD (Section 3.1) over the whole set of trajectories were: D_MEAN_ = 21.1 mm; SD = 32.5 mm; MAD = 56.3 mm.

**Figure 9:**
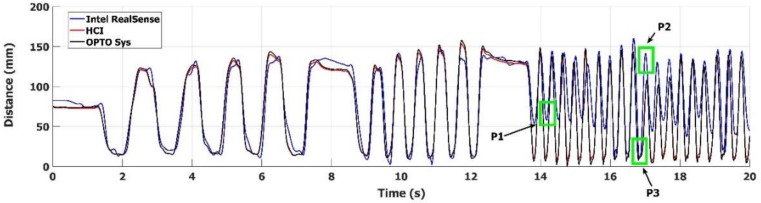
Comparison of the Intel RealSense^®^ and the HCI trackers during the execution of FT task movements. The figure shows the estimated distance between the thumb and the index fingers measured by the Intel RealSense^®^ tracker (blue solid line), the HCI tracking algorithm (red solid line) and the optoelectronic reference system (black solid line).

A typical result of the FT task performance execution is shown in Figure 9, where the distance between the index and thumb fingertips is plotted. The accuracy of the SR300 tracking firmware as compared to the HCI and to the optoelectronic reference system is clearly limited, especially in the period from 14 to 20 s, where the SR300 tracked distance shows large errors as compared to the distance measured by the HCI and the reference system. This period corresponds to high-speed FT movements, where in many cases the closing distance in the FT cycle does not correspond to a true fingers closure (P1 in Figure 9), or the true maximum amplitude is missed (P2 in Figure 9).

**Figure 10:**
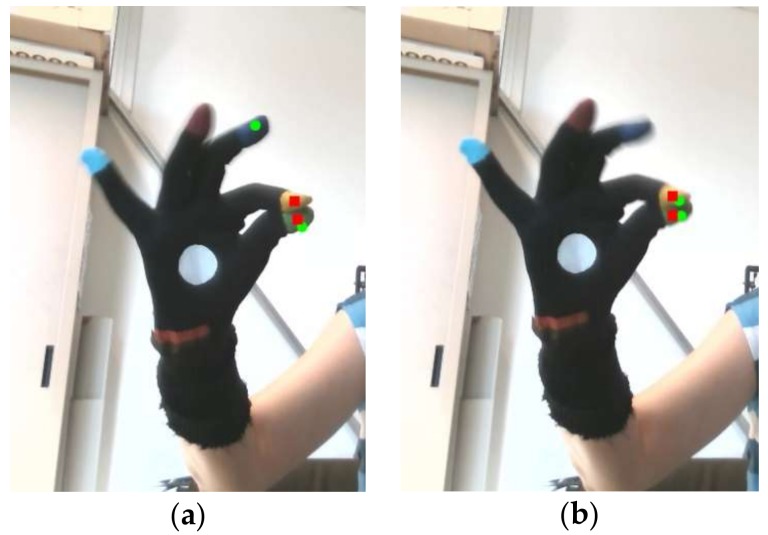
2D re-projections on RGB images of the 3D fingertip positions. Thumb and index from Intel RealSense^®^ tracker (green filled circles) versus thumb and index positions as estimated by the HCI tracker (red filled rectangles): (**a**) Incorrectly evaluated position of index finger by the Intel RealSense^®^ tracker (joint on middle finger, P1 in Figure 9); (**b**) Correctly evaluated position of the index finger for Intel RealSense^®^ tracker (P3 in Figure 9).

These problems are emphasized in Figure 10, where the 3D fingertip positions are re-projected on the SR300 RGB images. The incorrectly estimated closure of the peak P1 in Figure 9 is highlighted in Figure 10a, where the re-projected position of the index fingertip is incorrectly assigned to the middle fingertip (upper green filled circle). Some instability in the tracking of the hand model is apparent by comparison of Figure 10a,b, where two quite similar hand poses, corresponding to the peak P1 and P3 in Figure 9, gives a wrong distance estimation in Figure 10a and a correct one in Figure 10b.

### 5.2. Selection of Discriminant Kinematic Parameters

The parameter selection procedure retains those kinematic parameters which best correlate with neurologist UPDRS scores of the FT, OC and PS tasks. The results of the selection are shown in Table 3, Table 4 and Table 5 for the FT, OC and PS tasks, respectively. The parameter labels, the parameter meaning and the Spearman’s ρ values and sign of the correlation are shown in columns 1, 2 and 3, respectively. The good correlation of the selected parameters with the UPDRS scores is an important requirement for the automated assessment.

**Figure 11:**
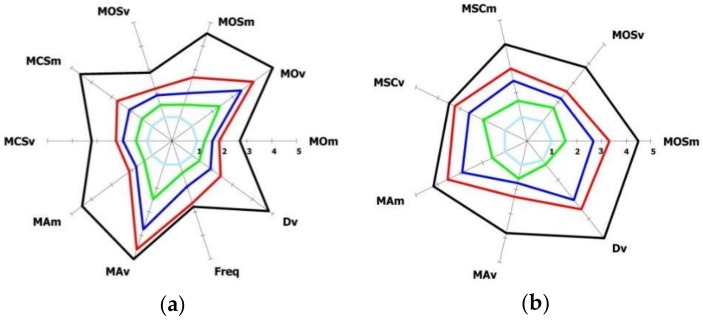

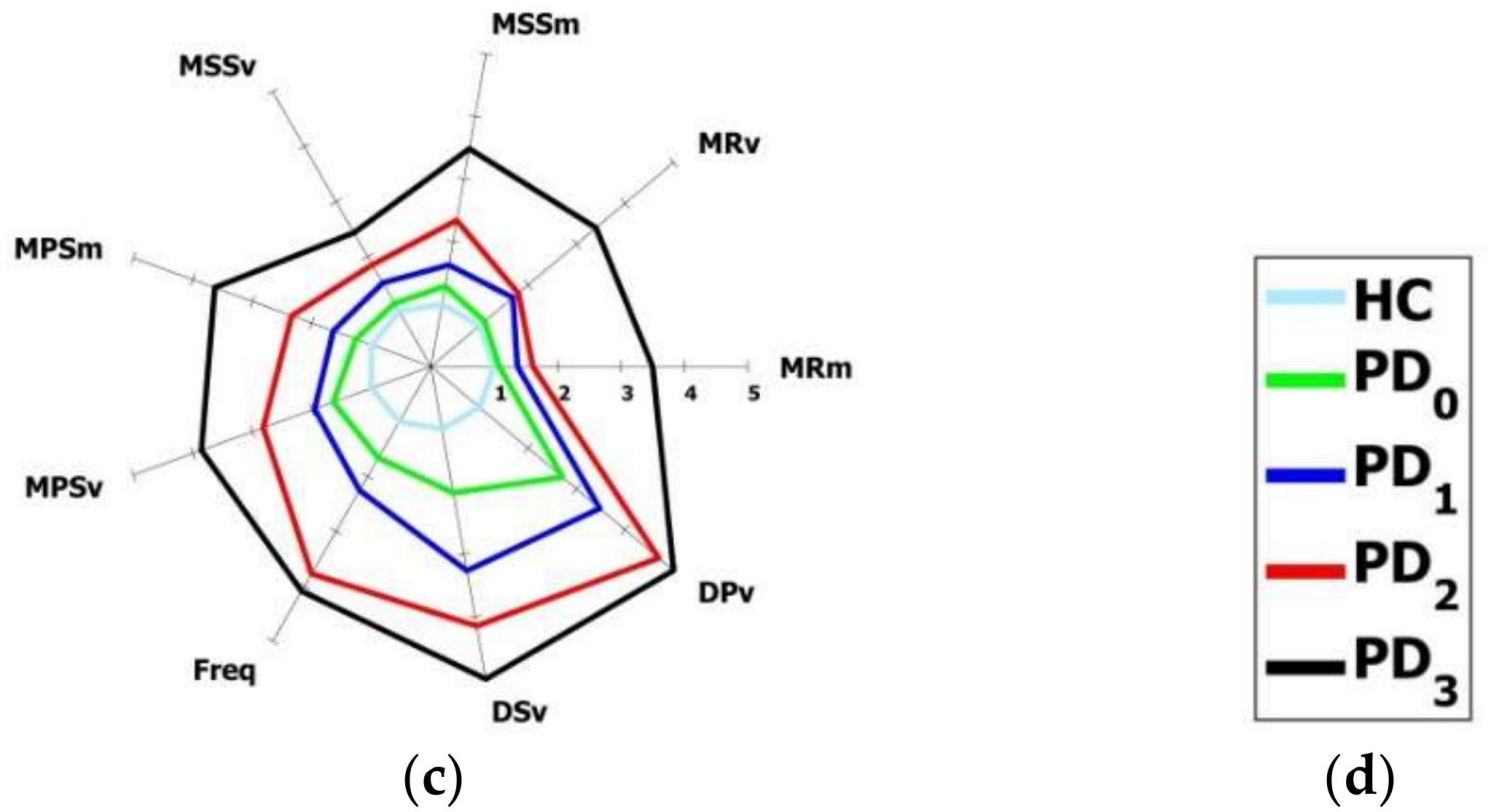
Radar plots of the mean values of the normalized kinematic parameters vs. the UPDRS severity class for the three upper limbs tasks: (**a**) Finger Tapping; (**b**) Opening-Closing; (**c**) Pronation-Supination. (**d**) Radar plots legend with HC and PD severity classes.

The mean values of the kinematic parameters versus the UPDRS severity class are shown in the radar graphs of Figure 11. The parameters were chosen such that increasing values indicate a worsening of the performance, which is visualized as a corresponding expansion of the related graph. As can be seen, almost all the selected parameters discriminate between different UPDRS severity classes. The different graphs are encapsulated and do not overlap, which means that, on the average, a monotonic increasing of the parameter value correspond to an increasing (i.e., worsening) of the UPDRS score.

### 5.3. Accuracies of the Supervised Classifiers in UPDRS Task Assessment

The results of the preliminary comparison among different supervised learning methods are shown in Table 6, in which classification accuracies, resulting from the leave-one-out and 10-fold cross validation methods for binary and multiclass classification problems, are reported. The “HEALTHY vs. PD” columns refer to the binary classification problem (healthy versus parkinsonian subjects), while “HEALTHY vs. UPDRS” columns refer to the five-classes classification problem (healthy subjects versus UPDRS scores of parkinsonian subjects). The obtained results suggested the use of SVM, not only for an overall greater mean accuracy, but also for the ability to limit the classification errors to one UPDRS score, unlike the other methods that sometimes generated classification errors greater than one UPDRS score. This is also an important requirement for obtaining the best agreement with the standard neurological assessment.

As a consequence of the preliminary comparison activity and focusing on the automated assessment of the UPDRS tasks, after the training by experimental data, the SVM supervised classifiers for the FT, OC and PS tasks have been validated by the leave-one-out cross validation method only on the PD cohort (four classes). The absolute classification error *e_c_* = |*C_i_* − *C*′*_i_*|, defined as the difference between the UPDRS class *C* assigned by the neurologist and the estimated UPDRS class *C*′, was never greater than 1 UPDRS class for all the tasks. Furthermore, the mean value of the error over the patients’ cohort for FT, OC and PS task was 0.12, 0.27 and 0.60, respectively. The classification performance of the classifiers has been evaluated by their confusion matrices and expressed concisely in terms of accuracy, defined as the number of true positives plus the number of true negatives, divided by the total number of instances. In our experiment, we used multi-class classifiers trained on almost balanced classes. In this case, the per-class accuracy, where the class classification accuracies are averaged over the classes, is more appropriate [48]. The resulting accuracies values obtained by the cross-validation methods for the FT, OC and PS classifiers are 76%, 65% and 58%, respectively.

## 6. Discussion

In this paper, a self-managed system for the automated assessment of Parkinson’s disease at home is presented. The core of the system is a low-cost non-invasive human computer interface which provides both a gesture-based interaction for the self-management of the task executions and, at the same time, the characterization of the patient movements by an accurate hand tracking.

Tracking accuracy is important because the automated assessment makes use of the correlation existing between kinematic parameters of the hand movements and the severity of the impairment.

### 6.1. Accuracy Comparison of the HCI Tracker Respect to Commercial Devices

We compared the accuracy of our HCI tracker with possible alternatives of widely used consumer hand tracking devices such as LMC and the Intel RealSense SR300.

The results of the comparison highlight some problems with these tracking devices. The accuracy of LMC in hand movement tracking is about ten times worse with respect to the HCI in the working volume, as shown in Table 1 and Table 2 also in the trajectory example of Figure 7 and Figure 8. In particular, the maximum absolute difference MAD has a considerably large value; this is probably due to some inconsistency in the tracking, which occurs randomly, as shown in Figure 8.

These results are consistent with those reported in [31,32] and in contrast to previous studies [28,29]. The fingertip speed is expected to increase from FT to OC movements and to reach maximum values for PS. A decrease in accuracy with speed is expected, and confirmed by the accuracy values of Table 1 and Table 2 for both the HCI and the LMC, which become worse as movement speed increases. The accuracy of proprietary hand tracker of the SR300 in the working volume for FT movement is even worse respect to the HCI; amplitude attenuations and missed closures of the fingertips are present, as shown in Figure 9. For conciseness reasons, we averaged the accuracy values for all the hand trajectories in the working volume, but considering the not aggregated values, we noted a worsening of the accuracy for the performances whose hand position is far from the device, as expected. 

Between the two devices, LMC is, at the moment, the only device able to track high-speed movements, but unexpected offsets and attenuations in the tracked trajectories are present and, for the most challenging high-speed movements of the PS task, severe inconsistencies on the fingertip and hand pose estimation occur. This is not unexpected, since the device is intended for general purpose applications, mainly in VR environments, while our tracking application is specialized on specific high-speed hand movements. On the other hand, the trajectories tracked by our HCI are satisfyingly close to those of the optoelectronic reference. 

Another problem with LMC is the working volume, which is very limited (0.08 m^3^) respect to the HCI one (0.45 m^3^); the user is forced to perform the motor tasks in a constrained environment, and this is an important limitation for the usability of the device by motor impaired people.

Nevertheless, care must be taken in extrapolating these accuracy results to more general tracking applications; effects on accuracy due to the several infrared sources present in the experiment have been experimentally evaluated, but more systematic work is necessary to exclude any interference. Furthermore, the typical short product life span of these consumer tracking devices and of the related software support rise concerns on their widespread use. Intel’s recent decision to discontinue the hand tracking firmware development for RealSense camera family is an example. Even if this decision has no impact on our PD assessment system, this is one more reason not to rely on solutions too dependent on closed and proprietary hand tracking firmware.

### 6.2. Kinematic Parameter Selection

A second goal of this work was the selection of the kinematic parameters of the hand movements which best correlate with clinical UPDRS scores. The Spearman non-parametric rank correlation was adopted to make the selection more robust to possible non-linear relationship between scores and parameters. The choice for this preliminary experiment to employ only one rater was dictated by the reason to not introduce, in the training datasets, different biases and noise due both to inter-rater disagreement and to the different sensitivity of the raters to specific motor aspects. The final choice for the best parameters was effective, as the radar plots of Figure 11 show, but heuristic, being based on the threshold on the Spearman correlation value combined with the visual evidence of discriminant power of the parameters on the radar plots. This discriminative power is different for each parameter; Freq for FT, or MRv and MRm for PS seem less discriminant than others when differentiating among the UPDRS classes (Figure 11), but only the last two have low correlation values. This indicates that the radar graphs catch only a part of the interdependence between UPDRS scores and parameters. Among the aspects not yet explored in this work, there is the integration of the assessments from more than one neurologist in the training set, and the pruning of some parameters of the selected set that are highly correlated each other.

### 6.3. Automated Assessments by Supervised Classifiers

The third goal of this work was the automation of the assessment by means of supervised classifiers trained on the selected parameters and the related UPDRS scores. SVM classifiers were chosen for their better performance on our training dataset with respect to the other types of classifiers we tested. We compared our results for classification accuracy with the results of some recent studies, even if many of them reported results only for the FT task and for binary classification (Healthy vs. Parkinsonian, HvsP). In particular, in [11], the UPDRS classes of the PD cohort were grouped, reducing the classification from multi-classes to a binary classification and declaring an accuracy for FT from 87.2% to 96.5%, depending on the grouping strategy. In [13], the HvsP binary classification was addressed, reporting a lower classification accuracy for FT (95.8%) with respect to our results, but on a wider cohort. In the same study, the results for the multi-classes classification are not directly comparable, since the classification was reduced to a 3-classes problem, resulting in a greater average classification accuracy for the FT task (around 82%). The classification results in [16] are limited to the HvsP binary classification for all the upper limbs UPDRS tasks. However, only the maximum accuracy for each classifier was included, ranging from 71.4% to 85.7%. Finally, the HvsP binary classification was also addressed in [18], and the results were reported for each UPDRS task; in this case, the classification accuracies ranged from 87.5% for PS to 100% for FT, but on a very limited number of HC and PD subjects. In conclusion, our results concerning the HvsP binary classification accuracy are overall better than the results of the previous studies mentioned [13,16,18] (see Table 6). Moreover, our results also seem good for the multi-classification case (e.g., [13]), considering that our classifiers addressed more classes than other studies. The classification accuracies obtained for the FT, OC and PS assessment tasks indicate that the classification errors were limited to 1 UPDRS class at most, and well below, on average. This result is compatible with the inter-rater agreement values usually found among neurologists for these tasks. A limitation of the present approach is that the subjectivity of the neurology judgment influences the machine learning process and, as a matter of fact, the classifiers mimic a particular neurologist. By using one neurologist, we reduce the inter-rater disagreement “noise” generally present in the training data of two or more neurologists but, of course, we reduce also the generalizing capabilities and the robustness of the automated assessment. Further work is required both to increase the training data set with the contribution of other neurologists and to harmonize their assessments. An important difference between the assessments of the system and those of the neurologists is that the system assesses the same motor performance with the same score, and do not show intra-rater disagreements. Summarizing, the results indicate that automated assessments of the upper limb tasks replicates the clinical ones, demonstrating its effectiveness in monitoring of PD at home. The present work is part of a project aimed at bringing the automated assessment of many UPDRS items into the home, for a more comprehensive assessment of the neuro-motor status of PD patients.

## 7. Conclusions

In this paper, a self-managed system for the automated assessment of Parkinson’s disease at home is presented. The automated assessment is focused on upper limb motor tasks as specified by standard assessment scales. The core of the system is a low-cost human computer interface which provides gesture-based interaction for the self-management of the task executions and an accurate characterization of the patient movements by selected kinematic parameters. The hand tracking accuracy of the system has been compared favorably with popular consumer alternatives.

The correlation between selected kinematic parameters and clinical UPDRS scores of patient performance has been used for the automated assessment by a machine learning approach based on supervised classifiers. The classifiers were trained by the assessments collected by the system and by a neurologist on cohorts of PD patients in an experimental campaign. The results on trained classifier performance show that automated assessments of the system replicate clinical ones, demonstrating its effectiveness. Furthermore, the system interface allows gestural interactions with visual feedback, providing a system management suitable for motor impaired users in home monitoring of Parkinson’s disease.

## Figures and Tables

**Table 1:** Accuracy of the HCI for FT, OC and PS movements.

Accuracy Parameters	FT	OC	PS
D_MEAN_ (mm)	2.5	3.1	4.1
SD (mm)	3	3.5	4
MAD (mm)	5.2	6.0	7

**Table 2:** Accuracy of the LMC for FT, OC and PS movements.

Accuracy Parameters	FT	OC	PS
D_MEAN_ (mm)	11.7	13.8	20.1
SD (mm)	13.3	22.8	26.6
MAD (mm)	32.1	35.2	45.1

**Table 3:** List of significant parameters for Finger Tapping task.

Name	Meaning	Spearman Correlation Coefficient ρ
MOm	Mean of Maximum Opening	−0.45
MOv	Variability ^1^ of Maximum Opening	0.32
MOSm	Mean of Maximum Speed (opening phase)	−0.57
MOSv	Variability ^1^ of Maximum Speed (opening phase)	0.36
MCSm	Mean of Maximum Speed (closing phase)	−0.58
MCSv	Variability ^1^ of Maximum Speed (closing phase)	0.40
MAm	Mean of Movement Amplitude	−0.44
MAv	Variability ^1^ of Movement Amplitude	0.38
Freq	Principal Frequency of voluntary movement	−0.46
Dv	Variability ^1^ of Movement Duration	0.44

^1^ Variability is equivalent to the coefficient of variation CV, defined as the ratio of the standard deviation σ to the mean μ, CV = σ/μ.

**Table 4:** List of significant parameters for Opening Closing task.

Name	Meaning	Spearman Correlation Coefficient ρ
MOSm	Mean of Maximum Speed (opening phase)	−0.61
MOSv	Variability ^1^ of Maximum Speed (opening phase)	0.42
MCSm	Mean of Maximum Speed (closing phase)	−0.58
MCSv	Variability ^1^ of Maximum Speed (closing phase)	0.56
MAm	Mean of Movement Amplitude	−0.57
MAv	Variability ^1^ of Movement Amplitude	0.34
Dv	Variability ^1^ of Movement Duration	0.55

^1^ Variability is equivalent to the coefficient of variation CV, defined as the ratio of the standard deviation σ to the mean μ, CV = σ/μ.

**Table 5:** List of significant parameters for Pronation Supination task.

Name	Meaning	Spearman Correlation Coefficient ρ
MRm	Mean of Movement Rotation	−0.30
MRv	Variability ^1^ of Movement Rotation	0.31
MSSm	Mean of Maximum Speed (supination phase)	−0.48
MSSv	Variability ^1^ of Maximum Speed (supination phase)	0.36
MPSm	Mean of Maximum Speed (pronation phase)	−0.44
MPSv	Variability ^1^ of Maximum Speed (pronation phase)	0.43
Freq	Principal Frequency of voluntary movement	−0.43
DSv	Variability ^1^ of Supination Duration	0.34
DPv	Variability ^1^ of Pronation Duration	0.35

^1^ Variability is equivalent to the coefficient of variation CV, defined as the ratio of the standard deviation σ to the mean μ, CV = σ/μ.

**Table 6:** Accuracies of classifiers for cross validation method and classification test.

		HEALTHY vs. PD		HEALTHY vs. UPDRS	
Task	Classifier	Leave-One-Out	10-Fold ^1^	Leave-One-Out	10-Fold ^1^
FT	NB	91.19	91.70	59.94	59.45
	LDA	93.71	93.71	66.31	66.63
	MNR	95.60	95.60	73.35	73.06
	SVM	98.23	98.44	76.06	76.71
	KNN	93.71	94.10	69.69	69.22
OC	NB	86.67	86.16	58.19	58.84
	LDA	88.57	88.57	61.05	61.56
	MNR	90.48	91.44	65.95	66.21
	SVM	90.48	90.06	65.14	65.24
	KNN	89.52	90.34	59.14	59.17
PS	NB	98.97	98.97	56.67	56.79
	LDA	91.75	91.75	55.67	57.10
	MNR	98.97	98.70	56.79	56.51
	SVM	98.97	98.97	58.73	58.87
	KNN	98.97	97.94	57.82	58.25

**^1^** mean accuracy on 500 classification trials.
